# Risk factors of delirium in paediatric intensive care units: A meta-analysis

**DOI:** 10.1371/journal.pone.0270639

**Published:** 2022-07-08

**Authors:** Xuelian ZHU, Xiaoyan FENG, Jia LIN, Yanhong DING

**Affiliations:** 1 Department of Orthopedics, Wuxi Children’s Hospital, Wuxi, China; 2 Nursing Department, Wuxi Children’s Hospital, Wuxi, China; 3 PICU, Wuxi Children’s Hospital, Wuxi, China; Universidade de Sorocaba, BRAZIL

## Abstract

**Background:**

Delirium is a brain dysfunction syndrome, which children have a higher incidence. At present, there have been more and more studies and reports on delirium in paediatric intensive care unit, but there are some differences in the risk factor results among different studies. To better manage delirium, this study was performed.

**Objective:**

To integrate and clarify the risk factors for delirium in paediatric intensive care unit.

**Methods:**

CNKI, CBMdisc, Wanfang Data Knowledge Service Platform, VIP, PubMed, Embase, Cochrane Library, JBI and PsycInfo were searched for relevant literature. The study subjects were patients in PICU and literature was included according to the PICOS principle. Literature screening and risk of bias assessment were mainly completed by two researchers, and RevMan 5.3 software and Stata software were used for data analysis. The GRADE systerm was used to assess the quality of evidence.

**Results:**

A total of 10 studies were included, all in English, involving 4343 children. Within the GRADE system, 4 indicators were scored A, 1 indicators were scored B, and 3 indicators were scored C regarding evidence levels. Three studies analysed the influence of developmental delay on the occurrence of delirium in PICU, total sample size of which was 1823, and the results showed that the combined effect was statistically significant [OR = 3.34, 95%CI(2.46–4.53), Z = 7.75, P<0.001]; Five studies analysed the effects of mechanical ventilation on the occurrence of delirium in PICU, sample size of which was 1562, and the results showed that the combined effect was statistically significant [OR = 4.11, 95%CI(3.13–5.40), Z = 10.16, P<0.001]; Two studies analysed the effects of benzodiazepines on children developing delirium, sample size of which was 1635, and the results showed that the combined effect was statistically significant [OR = 5.05, 95%CI(3.65–6.97), Z = 9.83, P<0.001]; Two studies analysed the effects of anticholinergic drug use on children developing delirium in PICU, sample size of which was 1703, and the results suggested the combined effect was statistically significant [OR = 5.04, 95%CI (3.62–7.00), Z = 9.63, P<0.001]; Two studies compared the same age period, sample size of which was 1724 and the results showed that children 2–5 years old has a 48% incidence rate of delirium relative to children younger than 2 years old, and the combined effect was statistically significant [OR = 0.48, 95%CI(0.25–0.92), Z = 2.22, P = 0.030], children 5–13 years old has a 39% incidence rate of delirium relative to children younger than 2 years old, and the combined effect was statistically significant [OR = 0.39, 95%CI(0.26–0.59), Z = 4.43, P<0.001]. Two studies analysed the effects of PICU LOS on children developing delirium and the combined effect of PICU LOS on the occurrence of delirium in children in PICU was statistically significant [OR = 1.10, 95%CI(1.05–1.15), Z = 4.07, *P*<0.001].

**Conclusion:**

Developmental delay, mechanical ventilation, benzodiazepine use, anticholinergic use, age and PICU length of stay are independent risk factors for delirium in children in PICU. However, only a few articles were included in this study, which may lead to a certain bias and affect the analysing results. More large-sample, multicentre studies should be conducted to further explore and clarify the independent influencing factors of delirium in children in PICU and to provide guidance for clinical practice.

## 1 Introduction

### 1.1 Overview and research significance

Delirium is a brain disorder characterised by acute cognitive and arousal changes [1, 2] with a slow reaction, continuous excited movement, and emotional instability or insecurity as the main manifestations [3]. Children have a high incidence of delirium. In one study [4], a delirium rate of 69% was found in patients with a length of stay > 48 hrs (37% of the total sample). Delirium in children leads to adverse outcomes, such as complications, falls, prolonged hospital stay, and prolonged mechanical ventilation and even death [5, 6]. Delirium also makes it more likely that the child’s cognitive, emotional, and social abilities were impaired after discharge, which makes the caregivers suffering [2, 4, 7]. Traube et al. revealed that the medical expenses of children with delirium were almost four times those of children without delirium in the paediatric intensive care unit (PICU) [8]. At present, there is an increasing number of reports on paediatric delirium, but there are some differences in the results regarding risk factors across studies, and the cases are relatively scattered. To better manage delirium in the paediatric population, related studies on risk factors for delirium in children were reviewed, and through meta-analysis, influencing factors were evaluated. The final goal is to help clinical staff identify risk factors for delirium, screen high-risk children, and provide a timely reference for prevention and prognosis improvement.

### 1.2 Diagnosis or screening of delirium

To judge delirium, psychiatrists and clinicians can refer to the Diagnostic and Statistical Manual of Mental Disorders (DSM) [9], the gold standard for diagnosis. Additionally, some brief and valid screening tools have emerged for use in recent years, such as the Cornell Assessment of Paediatric Delirium (CAPD), the Sophia Observation Withdrawal Symptoms Paediatric Delirium (SOS-PD) scale, the Paediatric Confusion Assessment Method for the ICU (pCAM-ICU), and the Preschool Confusion Assessment Method for the ICU (psCAM-ICU). However, suitable subjects of these tools have some differences. The CAPD is used to detect delirium in PICU settings and consists of eight items. It has a sensitivity of 94.1% (95% confidence interval [CI]: 83.8–98.8%), a specificity of 79.2% (95% CI: 73.5–84.9%), and Cronbach’s α of 0.90 [10]. The pCAM-ICU, a cognitive tool requiring patient cooperation, is restricted to children older than 5, limited to patients with developmental delay, and requires extensive nurse training. The pCAM-ICU assesses fluctuations in mental status, attention, altered levels of consciousness, and disorganised thinking and has been validated with a sensitivity of 0.83 and a specificity of 0.99 in critically ill children aged 5 and older [11]. The PD-scale of the SOS-PD scale consists of 17 items used for early screening of PD in critically ill children. It has an overall sensitivity of 92.3% and a specificity of 96.5% compared to the psychiatrist’s diagnosis for a cut-off score ≥ 4 points. The Pearson coefficient between the PD scale and the CAPD is 0.89% (95% CI: 0.82–0.93). The intraclass correlation coefficient is 0.90 (95% CI: 82.7–99.4) [12]. The psCAM-ICU is a highly valid, reliable delirium instrument for critically ill infants and preschool-aged children, demonstrating a specificity of 91% (95% CI: 90–93), a sensitivity of 75% (95% CI: 72–78), a negative predictive value of 86% (95% CI: 84–88), a positive predictive value of 84% (95% CI: 81–87), and a reliability κ–statistic of 0.79 (0.76–0.83) [13].

### 1.3 Research status and progress regarding the risk factors for delirium in children

Due to a late start, research on the risk factors for delirium in critically ill children is limited. Univariate analyses comprise the majority of studies, while there is little research based on multivariable logistic regression analysis of delirium risk factors. The risk factors included in these studies and the results of the same risk factors vary. There is controversy, and the risk factors remain unclear. The existing research on the risk factors for paediatric delirium is still incomplete.

### 1.4 The pathophysiology of delirium

The causes and mechanism of delirium are complex and have not yet been elucidated. It has been hypothesised that systemic inflammation may lead to damage to blood-brain barrier integrity or the production of inflammatory products in the brain, giving rise to ischaemia and neuronal apoptosis [14]. The most common pathways associated with delirium development include acetylcholine and/or melatonin deficiencies; the overrelease of dopamine, norepinephrine and/or glutamic acid; and changes in 5-hydroxytryptamine [15, 16]. The level of neurotransmitters in the brain shifts in response to physiological stressors. Ultimately, brain decompensation or chemical changes cannot be controlled [17].

## 2 Materials and methods

We registered the meta-analysis on Prospero; the ID number is CRD4202020208219.

### 2.1 Inclusion and exclusion criteria

#### 2.1.1 Inclusion criteria

According to the PICOS principle:

P (patient or population): Patients in PICUs, which are hospital units providing continuous surveillance and care to acutely ill infants and children.
I/E (intervention/exposure): Exposure includes physiological factors, therapeutic factors, drug factors, age factors, and other susceptibility factors.
C (comparison/control): Individuals not exposed to some susceptibility factors above.
O (outcome): Delirium, which means a disorder characterised by confusion, inattentiveness, disorientation, illusions, hallucinations, and agitation.
S (study design): Three research types include case-control studies, cohort studies, and cross-sectional studies. Raw data in the literature provide odds ratios (ORs) or relative ratios (RRs) and 95% CIs, or can obtain data-converted OR values and 95% CIs.

#### 2.1.2 Exclusion criteria

① The study was a duplicate publication; and ② the original research is inaccessible in a variety of ways.

### 2.2 Literature retrieval strategy

We developed a literature search strategy. The search words were ‘Child */Paediatric */Paediatric */PICU/P*ediatric Intensive Care Unit’, ‘Delirium’, ‘Risk factor*/Relative factor*/risk*/Relative risk*/Influencing factor*’, and ‘Case–Control stud*/Cohort Stud*/Cross-sectional stud*/Observational Stud*’. We combined Boolean logic operators, major topic terms, and free words to search the China National Knowledge Infrastructure (CNKI), China Biology Medicine Disc (CBMdisc), the Wanfang Data Knowledge Service Platform, the China Science and Technology Journal Database (VIP), PubMed, Embase, Cochrane Library, Joanna Briggs Institute (JBI), and PsycInfo. We set the article publication time range as of September 2021. We browsed the references included in the retrieved literature and were prepared to carry out a secondary search if necessary.

### 2.3 Literature screening and quality evaluation

After downloading the titles, two researchers preliminarily eliminated duplicate studies, studies with inappropriate research content or methods, or subjects or types by looking at the titles and abstracts. Then, we downloaded the full texts of the remaining studies and read them carefully, excluding studies in which the full texts were inaccessible or did not meet the screening criteria. Two researchers assessed the quality of the full texts by referring to the Newcastle–Ottawa Scale (NOS). We used the Grading of Recommendations Assessment, Development and Evaluation (GRADE) system, an approach to judge the quality of evidence and strength of recommendations, to evaluate the quality of evidence for outcome measures. When disagreement appeared between the two researchers, other researchers in the group participated in the evaluation to determine the quality of the studies or evidence together. Any difference was discussed in the group until consensus was reached.

### 2.4 Statistical methods

We extracted the data from the original study and analysed them via RevMan 5.3 and Stata 15.0 software. We examined heterogeneity among the studies using the Cochrane Q test. If *P* ≥ 0.1 and *I*^*2*^ ≤ 50%, we considered there to be homogeneity among the studies, and we could use a fixed effects model; otherwise, the source of heterogeneity could be identified as much as possible through sensitivity analysis. If heterogeneity could not be eliminated, we adopted a random effects model, and used descriptive analysis if the heterogeneity was too large and the source could not be determined. Value variable data are shown by weighted mean difference (WMD) and 95% CI. Dicategorised variable data are presented in the form of ORs and 95% CIs. We used Egger’s test to check for publication bias.

## 3 Results

### 3.1 Literature retrieval results

**Fig 1:**
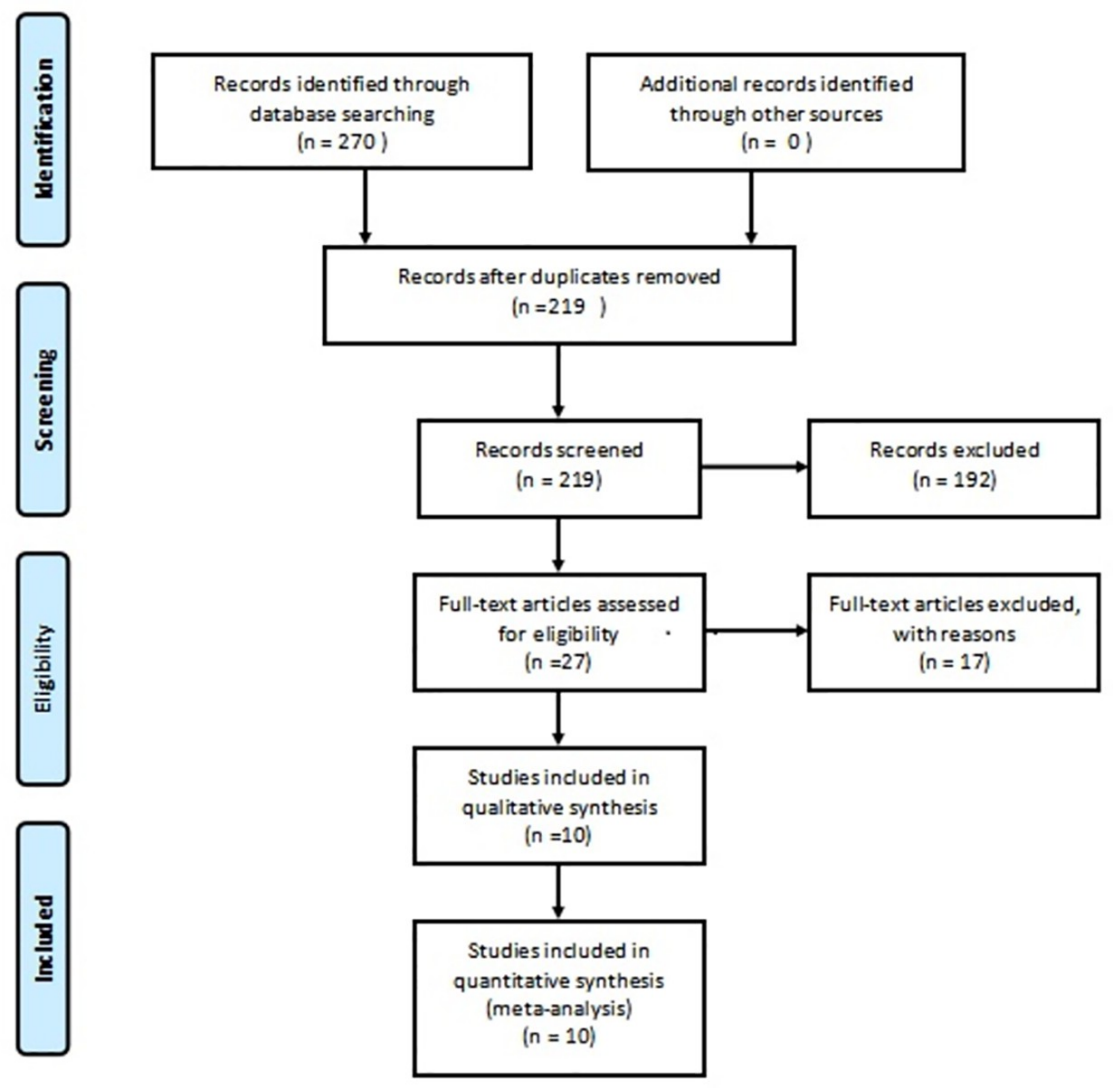
Literature retrieval and selection flowchart.

We initially searched a total of 270 studies, including 81 in Chinese and 189 in English. We screened out 51 duplicate studies and excluded 192 by reading the titles and abstracts due to unmatched research subjects or content or types; we initially included 27 studies. After further reading, we excluded 17 studies and finally included 10, all in English, encompassing a total of 4,343 patients (Fig 1).

### 3.2 Basic characteristics and methodological quality evaluation of the included studies (Tables 1–3)

**Table 1:** General characteristics of included studies.

Author	Published year	Type of research design	Research subjects	Number of delirium cases(sample)	Age(yr)	Gender	Source of subjects	Diagnostic tool	The risk factors involved
Silver G [18]	2015	Cohort study	Children	21 (99)	0–2 34% 2–5 19% 5–13 21% >13 25%	Boy 60.00% Girl 40.00%	A PICU	DSM-IV& CAPD	(1)(2)(3)
Traube C [19]	2017	Cohort study	Children in PICU	267 (1547)	0–2 38.27% 2–5 20.49% 5–13 22.62% >13 18.62%	Boy 56.88% Girl 43.12%	A PICU	CAPD	(1)(2)(3)(4)(5)(6)(7)
Alvarez RV [20]	2017	Cohort study	Children in CICU	56 (99)	0–1 45.46% 1–5 30.68% 6–12 12.5% 13–21 11.36%	Boy 48.86% Girl 51.14%	A PCICU	CAPD	(2)(3)(4)
Mody K [21]	2018	Cohort study	Children in PICU	131 (580)	0–1 25.3% 1–2 19.7% 3–5 19.7% 6–12 17.4% 13–21 17.9%	Boy 53.40% Girl 46.60%	A PICU	CAPD	(2)(4)(10)
Ramirez CR [22]	2018	Cohort study	Children(5–14 years) in PICU	29 (156)	5–14 100%	Boy 52.56% Girl 47.44%	A PICU	pCAM-ICU &DRS-R98	(2)(5)(11)(12)(13)(14)(15)
Patel AK [23]	2016	Cohort study	Children after heart bypass surgery	87 (177)	0–2 41.8% 3–5 24.7% 6–13 14.9% > 13 18.6%	Boy 58.8% Girl 41.2%	A PCICU	CAPD	(1)(3)(16)(17)(18)
Traube C_(1)_ [24]	2017	Cohort study	Children in PICU	209 (835)	0–2 48.7% 2–5 14.5% 5–13 19.9% > 13 16.8%	Boy 54.0% Girl 46.0%	25 PICUs in the US, the Netherlands, New Zealand, Australia, and Saudi Arabia	CAPD	(2)(3)(4)(19)(20)(21)(22)(23)
Ge X H [25]	2021	Case-control study	Children in PICU	200(639)	<0.5 292 0.5–1 145 1–3 87 >3 115	Boy 54.46% Girl 45.54%	Two PICUs	CAPD	(2)(3)(24)(25)(26)(27)(28)(29)
Yontem A [26]	2021	Cohort study	Children in PICU	14(142)	1.42–10.25 43	Boy 59.9% Girl 40.1%	A PICU	CAPD	(24)(30)
Staveski S L [27]	2021	Cohort study	Children in PICU that underwent cardiac surgery within the last 30 days	73(181)	2.50(mo,median)	Boy 51.38% Girl 48.62%	27 PCICUs in the USA	CAPD	(31)(32)(33)(34)(35)(36)

**Note:** Risk factors:(1)Developmental delay;(2)Mechanical ventilation;(3)Age;(4)Use benzodiazepines;(5)Use anticholinergic drugs;(6)Coma status;(7)Probability of mortality>1.4%;(8)Primary diagnosis of brain tumor;(9)Postoperative status;(10)Delirium status day prior;(11)Intellectual disability;(12)Hepatic failure;(13)Enfermedad neurológica;(14)Use other psychotropics;(15)Tachycardia;(16)Baseline albumin>3mg/dL;(17)Risk Adjustment for Congenital Heart Surgery;(18)Cyanotic heart disease;(19)Physical restraints;(20)Use narcotics;(21)Use antiepileptics;(22)General anesthesia;(23)Vasopressors;(24)LOS;(25)Hypoxia;(26)Metabolic dysfunction;(27)PRISM IV Score;(28)CRP;(29)Duration of Infection;(30)psychological intervention;(31)Pain score;(32)Total opioid exposure;(33)State Behavioral Scale score<0;(34)Pain medication or sedative administered in the previous 4 hours;(35)No progressive physical therapy or ambulation schedule in their medical record;(36)Parents not at bedside at time of data collection.

**Table 2:** Quality evaluation of studies.

The author	Year	Selection	Intergroup comparability	The results of measurement	NOS total score
Silver G [18]	2015	☆☆☆☆	☆☆	☆☆	8☆
Traube C [19]	2017	☆☆☆☆	☆☆	☆☆	8☆
Alvarez RV [20]	2017	☆☆☆☆	☆	☆	6☆
Mody K. [21]	2018	☆☆☆	☆	☆☆☆	7☆
Ramirez CR [22]	2018	☆☆☆☆	☆☆	☆☆	8☆
Patel AK [23]	2016	☆☆☆☆	☆	☆☆	7☆
Traube C_(1)_ [24]	2017	☆☆☆	☆	☆	5☆
Ge X H [25]	2021	☆☆☆☆	☆☆	☆☆	8☆
Yontem A [26]	2021	☆☆☆☆	☆	☆☆	7☆
Staveski S L [27]	2021	☆☆☆☆	☆	☆☆	7☆

**Table 3:** Assessment of evidence level based on GRADE system.

Risk factors (Number of studies)	Sample size	Limitations	Inconsistency	Indirectness	Imprecision	Publication bias	Effect size	Mixed effect	Dose-effect relationship	Quality of evidence
Developmental delay(3)	1823	/	/	/	/	/	+1	+1	/	A
Mechanical ventilation(5)	1562	/	/	/	/	/	+1	+1	/	A
Benzodiazepine use(2)	1635	/	/	/	/	-1	+1	/	/	A
Anticholinergic drug use(2)	1703	/	/	/	/	-1	+1	/	/	A
Age(0–2) &Age(2–5)(2)	1724	-1	/	/	/	-1	/	/	/	C
Age(0–2) &Age(5–13) (2)	1724	-1	/	/	/	-1	/	/	/	C
Age(0–2) &Age(>13)(2)	1724	-1	/	/	/	-1	/	/	/	C
PICU LOS(2)	781	/	/	/	/	-1	/	/	/	B

We included nine cohort studies and one case–control study, all published in the last 5 years. The subjects were children and adolescents not more than 21 years old, and all were from the PICU. The number of patients in each study ranged from 99 to 1,547. The risk factors included in the studies are displayed in Table 1. We performed quality evaluation based on the NOS and NOS total scores of the studies, all marked with 5 to 8 stars (Table 2). Within the GRADE system, 4 indicators were scored A, 1 indicator was scored B, and 3 indicators were scored C regarding evidence levels (Table 3).

### 3.3 Meta-analysis results

#### 3.3.1 The effects of developmental delay on PICU delirium

**Fig 2:**
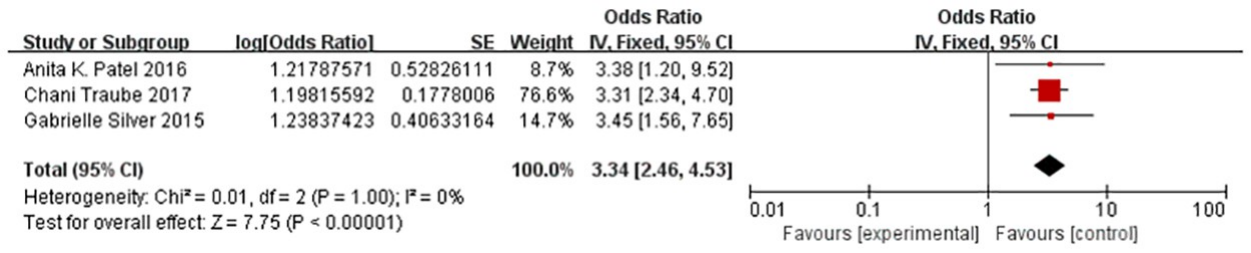
Forest plot for developmental delay analysis. We conducted meta-analysis for 3 studies using a random effect model. We used ORs and 95% CIs to represent the results.

Three articles included in this study examined the effects of developmental delay on the occurrence of delirium in children [18, 19, 24]. We did not detect obvious heterogeneity (*I*^*2*^ = 0%, *P* = 1.000), and we used a random effects model due to the potential heterogeneity among the studies. The combined effect was statistically significant [OR = 3.34, 95% CI (2.46–4.53), Z = 7.75, *P*<0.001], suggesting that developmental delay is an independent influencing factor for the occurrence of paediatric delirium (Fig 2). Publication bias was not apparent according to Egger’s test outcomes [*t* = 1.540, *P* = 0.366].

#### 3.3.2 The effects of mechanical ventilation on PICU delirium

**Fig 3:**
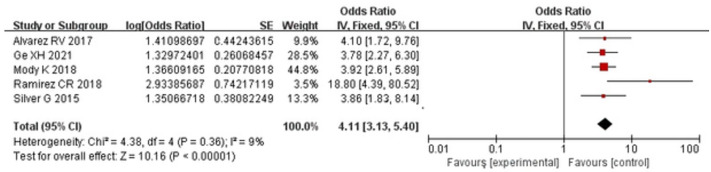
Forest plot for mechanical ventilation analysis. We conducted meta-analysis for 5 studies using a random effect model. We used ORs and 95% CIs to represent the results.

The effects of mechanical ventilation on paediatric delirium were discussed in seven articles [18–22, 24, 25]. The heterogeneity test suggested strong heterogeneity among the selected studies (*I*^*2*^ = 77%, *P*<0.001); the heterogeneity was still strong when we used a random effects model. Therefore, we employed sensitivity analysis to further explore the source of heterogeneity. After removing the selected studies one by one, the studies of Traube C 2017 [19] and Traube C 2017_(1)_ [24] were the main sources of heterogeneity, as they have the same first author and have a similar influence on the combined effect. We carried out descriptive analysis for 2 studies, and meta-analysis for 5 studies using a random effects model, with no obvious heterogeneity (*I*^*2*^ = 9%, *P* = 0.360) [OR = 4.11, 95% CI (3.13–5.40), Z = 10.16, *P*<0.0000] (Fig 3). We determined mechanical ventilation to be an independent risk factor for delirium in children. Traube C [19] showed that mechanical ventilation is an independent risk factor for delirium. Another study by Traube C [24] revealed the same conclusion. Publication bias was not apparent [*t* = 2.090, *P* = 0.104].

#### 3.3.3 The effects of benzodiazepine use on PICU delirium

**Fig 4:**
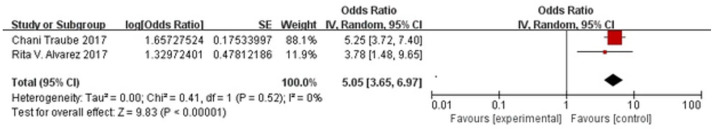
Forest plot for benzodiazepine use analysis. We conducted meta-analysis for 2 studies using a random effects model. We used ORs and 95% CIs to represent the results.

Three articles [19–21] examined the influence of benzodiazepines on children developing delirium. The analysis indicated that the heterogeneity among the studies was strong (*I*^*2*^ = 86%, *P* = 0.001). We removed the articles one by one and found that the main source of heterogeneity may have been Mody K 2018 [21], in which the considered factor was benzodiazepine exposure on the previous day, which was different from other studies on benzodiazepine exposure. After deletion, we performed meta-analysis for the remaining 2 articles, which demonstrated no obvious heterogeneity (*I*^*2*^ = 0%, *P* = 0.520); thus, we used a random effects model for analysis. The results signalled that benzodiazepine use is an independent risk factor for the occurrence of paediatric delirium, and the combined effect was statistically significant [OR = 5.05, 95% CI (3.65–6.97), Z = 9.83, *P*<0.001] (Fig 4). Mody K [21] illustrated that benzodiazepine use is closely associated with the transition from normal cognitive status to delirium, with a more than fourfold increase in the delirium occurrence rate.

#### 3.3.4 The effects of anticholinergic drug use on PICU delirium

**Fig 5:**
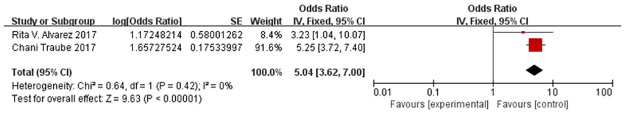
Forest plot for anticholinergic drug use analysis. We conducted meta-analysis for 2 studies using a random effects model. We used ORs and 95% CIs to represent the results.

We included two articles [19, 20] to observe the effects of anticholinergic drugs on the occurrence of childhood delirium. The heterogeneity test revealed no significant heterogeneity (*I*^*2*^ = 0%, *P* = 0.420), and we used a random effects model for analysis. The results imply that the combined effect of the independent risk factors regarding the occurrence of delirium in children treated with anticholinergic drugs is statistically significant [OR = 5.04, 95% CI (3.62–7.00), Z = 9.63, *P*<0.001] (Fig 5). Because we only included two articles in the analysis, we could not employ Egger’s test to detect publication bias, as doing so would have been meaningless.

#### 3.3.5 The effects of age on PICU delirium

**Fig 6:**
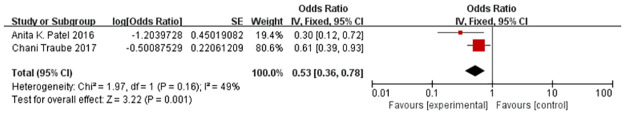
Forest plot for ages between 0 to 2 and 2 to 5. We conducted meta-analysis for 2 studies using a random effects model. We used ORs and 95% CIs to represent the results.

**Fig 7:**
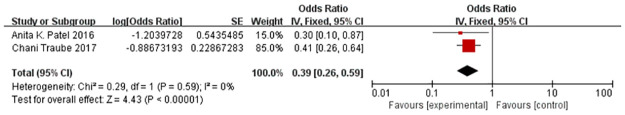
Forest plot for ages between 0 to 2 and 5 to 13. We conducted meta-analysis for 2 studies using a random effects model. We used ORs and 95% CIs to represent the results.

**Fig 8:**
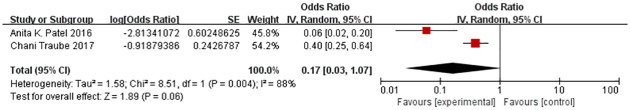
Forest plot for ages between 0 to 2 and > 13. We conducted meta-analysis for 2 studies using a random effects model. We used ORs and 95% CIs to represent the results.

The effects of age on the occurrence of paediatric delirium were examined in 6 articles [18–20, 23, 24]. We used descriptive analysis for 3 articles of different age groups, and we performed a meta-analysis for the remaining 3 articles [18, 19, 23]. We noted significant heterogeneity via a heterogeneity test (*I*^*2*^ = 85%, *P*<0.001) in the analysis between children aged 0 to 2 and children aged 2 to 5 among 3 articles. We used sensitivity analysis to scrutinise the sources of heterogeneity; the main source of heterogeneity might be found in the work of Silver G [18], as the number of cases was relatively small. After removing four studies, we compared children aged 0 to 2 with those aged 2 to 5, 5 to 13, and > 13 in the remaining 2 studies to identify the effects of age on paediatric delirium. These comparisons showed low heterogeneity, no obvious heterogeneity, and significant heterogeneity (*I*^*2*^ = 49%, *P* = 0.160; *I*^*2*^ = 0%, *P =* 0.590; I^2^ = 88%, P = 0.004); thus, we used a random effects model for analysis. Children aged 2 to 5 had a 48% incidence rate of delirium relative to children younger than 2, and the combined effect was statistically significant [OR = 0.48, 95% CI (0.25–0.92), Z = 2.22, *P =* 0.030]. Children aged 5 to 13 years old had a 39% incidence rate of delirium relative to children younger than 2, and the combined effect was statistically significant [OR = 0.39, 95% CI (0.26–0.59), Z = 4.43, *P*<0.001]. The children (>13 years old) and (<2 years old) displayed no significant difference [Z = 1.89, *P* = 0.060] (Figs 6–8). Alvarez RV [20] revealed that age is an independent risk factor for delirium and indicated that the probability of delirium decreases by 65% with each additional month of age. In addition, Traube C_(1)_ [24] demonstrated that age (> 2 years old) is an independent, protective factor against delirium.

#### 3.3.6 The effects of PICU LOS on PICU delirium

**Fig 9:**
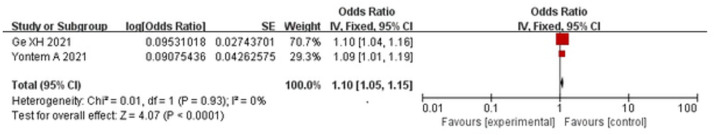
Forest plot for PICU LOS analysis. We conducted meta-analysis for 2 studies using a random effects model. We used ORs and 95% CIs to represent the results.

We included two articles [25, 26] to observe the effects of PICU LOS on the occurrence of childhood delirium. The heterogeneity test indicated no significant heterogeneity (*I*^*2*^ = 0%, *P* = 0.420), and we used a random effects model for analysis. The combined effect of PICU LOS on the occurrence of delirium in children in the PICU was statistically significant [OR = 1.10, 95% CI (1.05–1.15), Z = 4.07, *P*<0.001] (Fig 9).

## 4 Discussion

### 4.1 Methodological quality evaluation of the included literature

The number of studies included in the analysis was relatively small. All 10 studies were above 5 stars for NOS scores. The evidence levels of the study indicators were medium or high. The study indicators were relatively concentrated, and the meta-analysis showed good intergroup homogeneity. The subject sources and the inclusion and exclusion criteria were all clear.

### 4.2 Main findings and implications for clinical practice

The possible factors for the incidence of delirium in children were developmental delay, mechanical ventilation, benzodiazepine use, anticholinergic drug use, and age. The systematic review performed by Holly C et al. [28] revealed that young age, male sex, developmental delay, mechanical ventilation, and anxiety factors contribute to a higher likelihood of developing delirium, which is partly consistent with our results. The ORs and 95% CIs were not part of the work of Dervan LA et al. [29]; therefore, we did not include them in this study. This result suggests that delirium is independently correlated with age (< 2 years old) and mechanical ventilation, corresponding to our findings. This study can guide healthcare providers to address particular aspects, enhance their understanding of paediatric delirium, and help them to prevent delirium. We found that children with developmental delay are 3.34 times more likely to develop delirium than those with normal development. The probability of delirium in children on mechanical ventilation is 4.11 times higher than in children without mechanical ventilation. Children who have used benzodiazepines have a 5.05 times greater risk of delirium than those who do not, and children who use anticholinergic drugs are 5.04 times more likely to develop delirium than those who do not. Age and PICU length of stay are also independent risk factors for delirium. Hence, healthcare providers should pay more attention to children with developmental delay and conduct interventions to promote their growth processes. For children on mechanical ventilation, indications for machine withdrawal should be evaluated daily, and basic care should be well maintained to help children get offline as soon as possible. Benzodiazepine and anticholinergic drug use need to be balanced. Common PICU anticholinergic drug were all low-level, with midazolam (the most common) in the highest anticholinergic drug scale scores (95%), followed by vancomycin(81%), piperacillin (79%), and morphine (74%) [30]. Alternative drugs should be considered in patients requiring large amounts of anticholinergic burden drugs, and further screening and assessment should be made using validated scales for anticholinergic exposure [31]. Children younger than 2 years old also need attention. Under the premise of ensuring safety, the time spent in the PICU is reduced as much as possible. By strengthening the management of delirium risk factors, the occurrence of delirium can be mitigated. Timely reporting and appropriate interventions are critical.

### 4.3 Limitations

There was a small number of included studies, mainly carried out in European and American countries, which may lead to a certain bias in the analysis and affect the results.

### 4.4 Future outlook

This study suggests that healthcare providers need to strengthen the identification of risk factors and actively draw up care plans based on the above factors and implement them. However, challenges include variable delirium symptoms, a lack of specialised training, and low compliance rates for performing assessments [17]. According to a survey [32], although delirium is common in critically ill children, only 2% of PICUs screen for delirium. As such, it is significant to develop an appropriate delirium evaluation procedure or criterion [7]. A previous study [33] found that nurses in the PICU lacked an understanding of risk factors. In another study, 42 PICU nurses were trained, and knowledge of and attitudes toward delirium were significantly improved after training [26]. Nurses play an important role in helping with the prevention and care of delirium. It is necessary to do some training. The training content can involve the diagnosis, evaluation and identification of delirium, adverse consequences, and risk factors. For the prevention and treatment of delirium, non-pharmacological interventions such as music therapy, massage, avoidance of acousto-optic stimulation, exercise, and family participation should be emphasised [4].

## 5 Conclusion

Paediatric delirium has a high incidence rate; hence, there is a need to explore the risk factors for delirium and to strengthen the screening of delirium by improving clinical staff cognition to effectively prevent delirium. We found that developmental delay, mechanical ventilation, benzodiazepine use, anticholinergic use, age, and PICU length of stay are independent risk factors for delirium in paediatrics. However, we only included a few studies in this meta-analysis, which may have resulted in a certain degree of publication bias. Consequently, more large-sample, multicentre studies should be conducted to further explore and clarify the independent influencing factors of delirium in paediatrics and to provide guidance for clinical practice.

## Supporting information

S1 Table Checklist of items to include when reporting a systematic review or meta-analysis. (DOCX)

## Abbreviations

CAPD: the Cornell Assessment of Pediatric Delirium
CBMdisc: China Biology Medicine disc
CICU: Cardiac Intensive Care Unit
CNKI: China National Knowledge Infrastructure
GRADE: Grades of Reccommendations Assessment, Development and Evaluation
JBI: Joanna Briggs Institute
LOS: Length of stay
NOS: Newcastle-Ottawa Scale
OR: Odds Ratio
pCAM-ICU: the Pediatric-Confusion Assessment Method for the Intensive Care Unit
PCICU: Paediatric Cardiac Intensive Care Unit
PICU: Paediatric Intensive Care Unit
psCAM-ICU: the Preschool Confusion Assessment Method for the ICU
SOS-PD scale: the Sophia Observation Withdrawal Symptoms scale Pediatric Delirium scale
VIP: China Science and Technology Journal Database
