# Supplementary figures and images for: Risk factors of delirium in paediatric intensive care units: A meta-analysis
Source: PLoS One. 2022 Jul 8;17(7):e0270639. doi: 10.1371/journal.pone.0270639 (PMC9269883; doi:10.1371/journal.pone.0270639)

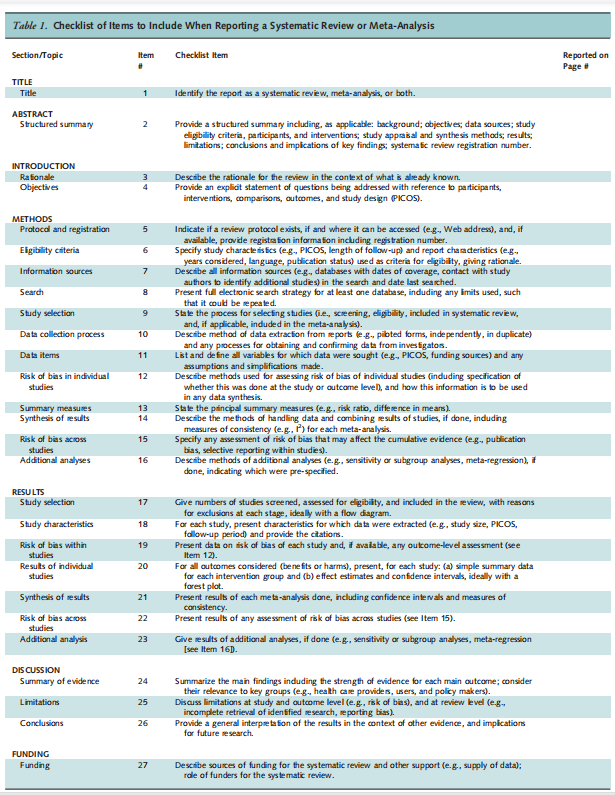


3-4

4

9

NA

9-10

8-9

6-8

6-8

6-8

6-8

6-8

4-5

4

4

4

4

4

4

4

3-4

3-4

3

3

2

2-3

1-2

1

Supplement: S1 Table — (DOCX) [file pone.0270639.s001.docx]
